# Feasibility and Acceptability of an Online Mindfulness-Based Intervention for Stress Reduction and Psychological Wellbeing of University Students in Pakistan: A Pilot Randomized Controlled Trial

**DOI:** 10.3390/ijerph20085512

**Published:** 2023-04-14

**Authors:** Anum Sarfraz, Salma Siddiqui, Julieta Galante, Siham Sikander

**Affiliations:** 1 Department of Behavioral Sciences, School of Social Sciences and Humanities, National University of Sciences and Technology, Islamabad 44000, Pakistan; 2 Department of Psychiatry, University of Cambridge, Cambridge CB2 0SZ, UK; 3 Department of Primary Care and Mental Health, University of Liverpool, Liverpool L69 3GF, UK

**Keywords:** mindfulness, stress, young adults, psychological wellbeing

## Abstract

The rise in mental health concerns of university students is causing a serious hinderance to their wellbeing, impeding their functioning. The socio-economic and political friction in low- and middle-income countries adds to their vulnerability and calls for a cost-effective indigenous intervention. Consequently, this study aimed to inform a large definitive trial by assessing the feasibility and acceptability of a randomized controlled trial (RCT) design evaluating a culturally adapted online Mindfulness Training Course (MTC) used to improve stress and wellbeing among Pakistani university students. A two-arm pilot randomized controlled trial was conducted. University students (*n* = 156) were randomly assigned to either the MTC group (*n* = 80) or Wait-list (WL) control group (*n* = 76) and completed baseline and post-intervention self-report measures for mindfulness, stress and psychological wellbeing. Additionally, semi-structured interviews were conducted with consenting MTC group participants (*n* = 18) to explore their views about MTC, employing reflexive thematic analysis. Of 80 participants randomized to the MTC group, 32 completed the course, whereas, from the 156 randomized participants, 102 completed assessment surveys. Feasibility and acceptability indicators showed high recruitment, compliance, and adherence to MTC, with practical steps for randomization and online data collection. Further results showed higher levels of mindfulness and psychological wellbeing and lowered stress levels in the MTC group compared to the control group. The attrition and dropout rates were high; however, the feedback from participants who completed the MTC was highly positive and encouraging. In conclusion, if the trial proceeds with increased outreach in a large-scale RCT, the recruitment might be revised to reduce attrition rates. Further recommendations are discussed.

## 1. Introduction

Mindfulness-based interventions (MBI) have shown benefits in improving the mental health of university students. The empirical evidence indicates their usefulness in enhancing wellbeing [1], resilience [2], stress [3], and academic performance [4,5]. The benefits have been shown to go beyond academic life and into young adults’ everyday life improving their social and personal lives [6]. The purpose of educational institutions is to foster individuals who are functional beings beyond academics [7], and MBIs seem to provide that space with their non-stigmatizing, non-critical, non-judgmental, accepting, present-oriented focus [8] adding to their high acceptance among students [9]. In a high-quality systematic review including fifty-one randomized controlled trials (RCTs), Dawson and colleagues [10] found that MBIs produced small to moderate effect sizes in decreasing distress, anxiety, depression, rumination and increasing wellbeing, and dispositional mindfulness in university students. The studies found by Dawson and colleagues were primarily conducted in the USA, which has a particular institutional culture different from other countries [11]. The review also highlighted the variability among MBIs and the lack of sufficient detail regarding intervention and delivery procedures, which significantly impede the application of MBIs in different cultures and countries. In general, there is a considerable lack of systematic approach to developing, adapting, and implementing mindfulness-based interventions [12,13]. Despite the increasing MBI research in the past few decades, methodological limitations hinder the implementation and utility in not only different countries but in the same country and settings as well.

The effects of MBIs in low- and middle-income countries (LAMICs) have also been studied through RCTs, indicating small to moderate effect sizes for emotional intelligence [14], perceived stress [15,16], and quality of life [17], in university students. The quality of these trials in LAMICs is still in its infancy regarding the research design and methodological rigor. For example, Chiodelli and colleagues [18] found increased levels of mindfulness and emotion regulation in Brazilian university students at post-intervention assessment after a 6-week adapted version of the MBI “Finding peace in a frantic world” [19]. The absence of random assignment and control group are limitations that make the results from their work less conclusive, and the lack of adaptation procedures’ reporting makes them less applicable.

In the context of LAMICs, low-resource interventions are needed. Online mindfulness-based interventions are cost effective and may be beneficial [20]. However, these interventions have been either self-help, DVD-delivered, or website-delivered [21,22], in which live weekly interaction with a facilitator is not present. This lack of human interaction may make online interventions less effective, and there are ways in which a human interaction element could be added to online MBIs.

In Pakistan, rising mental health awareness has raised concern among university students, who have been reported to suffer from academic stress and mental health problems including depression, anxiety, and suicide attempts [23,24,25]. The added challenges of political instability, lowered access to professional help [26], and stigma towards mental health [27,28] point towards the need for an intervention that helps cultivate resilience in students and improve their wellbeing using their inner resources. Mindfulness helps cultivate the resource with acceptance and equanimity in a stigma free self-exploratory approach, and an online mode of delivery with facilitator can partially address the scarcity of mental health services. Despite the prevailing mental health concerns, to our knowledge, there has been no research on mindfulness-based interventions in Pakistan.

In this context, we set out to culturally adapt an MBI that can be delivered online for Pakistani university students and has a human interaction element, and to test it in an initial pilot RCT. We ensured that systematic and methodological rigor was present [29]. We employed Medical Research Council Guidelines (MRC) for development of complex interventions [30]. We first conducted a systematic review of mindfulness practices in the LAMICs, which built the evidence base (CRD42019151902, manuscript in preparation). Next, the book “Finding Peace in a Frantic World” [19] was translated and adapted to address students’ stress and wellbeing, using the Heuristic framework for adapting interventions [31], involving stakeholders (teachers, students, and local and international mindfulness practitioners) under the supervision of an adaptation committee (clinical psychologist, researcher, and mindfulness practitioner). The adaptation indicated universality in core principles of intervention components. However, major surface structure changes included language, length of the reading material, metaphorical expressions, and an additional orientation audio recording. The adaptation process of “Finding Peace in a Frantic World” is detailed in another article [32]. Then, the adapted mindfulness-based intervention (called the Mindfulness Training Course—MTC) was piloted in a pre-posttest design on a group of students [33]. This manuscript reports the findings of the feasibility and the acceptability of the MTC for improving the stress and wellbeing of university students through a pilot RCT. There were four aims of the study with specific research questions. These were as follows:

(a) To inform a larger definitive trial by assessing:

Which of the four recruitment methods has more registrations?

What is the recruitment rate?

Is the online mode of delivering intervention sessions feasible?

Are the eligibility criteria of trial participants feasible?

Are the randomization procedures feasible?

Are online data collection procedures feasible?

Are worksheet submissions feasible?

Are the outcome measures being used feasible?

(b) To examine the acceptability of the trial:

Is the online mode of delivery (videoconferencing) acceptable? Is randomization acceptable to participants?

What is the attrition rate?

(c) To evaluate the acceptability of the intervention:

What is the dropout rate?

Is participant attendance maintained through the intervention?

Is home practice completed by participants?

What is the participants’ perceived acceptability of different elements of intervention, barriers, and facilitators to practice?

(d) To examine the mean difference in mental health outcomes (stress, psychological wellbeing, and mindfulness) between the two trial arms.

This is the first study to use questionnaires as well as interviews along with descriptive statistics to assess feasibility and acceptability of the MTC in a pilot RCT.

## 2. Methods

A convergent-parallel mixed methods randomized controlled pilot trial was conducted with an intended allocation ratio of 1:1 with a minor difference in that the MTC group had four more participants than the Wait-list group. The amendment in the protocol was based on participants’ request to shift from the wait-list group due to their unavailability during the scheduled MTC group weeks, hence the difference in both groups. The study was carried out online via Zoom videoconferencing and approved by the National University of Sciences and Technology’s ethics committee (0098/Ethic/01/S3H/008/DBS). The study protocol was prospectively registered at Clinicaltrials.gov (NCT05216445).

### 2.1. Participants and Recruitment

The volunteer students were recruited online using four modes. They attended different universities across Pakistan. The universities were identified based on research assistants’ (RA) outreach resource. The four modes of recruitment, assessed for feasibility were as follows:

Social media: Non-official WhatsApp groups, Instagram, Twitter, Facebook, and LinkedIn accounts managed by students.

University platforms: Official university group pages and email groups.

Recommendations: Academics, alumni, and students referred other students who might benefit from the training.

Direct emails/messages: Emails and WhatsApp messages sent to students directly by RAs.

Two RAs not involved in any other part of the project were responsible for recruiting participants. A standard flyer was developed, and RAs were briefed about the project to address student concerns during recruitment. After completing recruitment, they submitted a report to the lead researcher (AS) describing their recruiting process (Table 1). The flyer contained the dates and duration of the training, the dates of weekly sessions, eligibility for enrolled students, weekly home tasks, and the provision of weekly training material. The active link for registration on the flyer had a consent form providing detailed information about the training, confidentiality, anonymity of data, and participants’ right to withdraw. The lead researcher’s (AS) official email address was provided in the flyer, with an active link.

Interested students were included provided they were (i) 18 years or older, (ii) enrolled in an undergraduate or postgraduate course in a university in Pakistan. The students were excluded if they reported (i) any current diagnosed severe mental illness, e.g., severe depression or psychotic illness, (ii) any current diagnosed severe medical illness, e.g., chronic pain, (iii) undergoing any form of psychotherapy/counseling or psychiatric medication. Selection criteria was self-reported. Reporting of mental health issues is subjective and self-reporting may have lowered reliability; however, in this study in a non-clinical population, the students were asked about concrete diagnoses rather than an assessment of symptoms.

The sample size for the trial was based on the most agreed-upon size for a pilot trial; *n* = 24 [34,35] and the high attrition rate in mindfulness studies [36,37] specifically online mindfulness interventions ranging from 8–60% [9,38]. As per the protocol, to retain a total sample size of *n* = 24 and assuming the attrition rate a = 0.5, the study aimed for a sample size of *n* = 48, i.e., 24 participants in each group. However, the protocol was amended due to the large number of enrollments in a short period of time (see Results for final figures). Hence, recruitment was not stopped when the target number (*n* = 24) was achieved. Recruitment continued for two weeks, after which all the registered participants were screened for eligibility through the registration form. The eligible participants were contacted to schedule the initial interview call with the course facilitator (AS). During the interview call, participants were briefed on the study, inquired about any missing information from the registration form, and informed about their randomly assigned group after verbally consenting to randomization.

The participants (*n* = 156) were randomized to either the MTC (*n* = 80) or WL (*n* = 76) group. Before the initial interview call, a randomizing researcher first generated a sequence using a computerized random number generator (each number treated as an ID) and then assigned an ID (1–156) alternatively to one of the groups based on the sequence in a sealed envelope. The sealed envelopes with only a random ID number written on them were given to the facilitator, who opened the envelope in the initial interview and informed the participants about their allocated groups (written inside the envelope). In this way, the course facilitator and the participants were blinded to the allotment of group at the time of assignment by the randomizing researcher; however, once revealed and informed both were aware of the assigned group. Complete double-blinded randomization was not possible due to nature of the training.

### 2.2. Procedure

Since registrations for the course were higher than anticipated, participants in the MTC group were divided into three batches for the weekly sessions (two on Saturday and one on Sunday) to accommodate all students over the weekend and retain sufficient individualized attention in online group sessions. This decision was made during the interviews: as the number of participants in one batch reached 25 (per protocol), the subsequent calls were assigned to subsequent batches (Saturday evening and Sunday morning).

After the recruitment, screening and allocation, both groups completed assessments at two time points. First, *baseline assessment*, including demographic questionnaire and mental health outcome measure questionnaires. Second, the *post-assessment* included the same mental health outcome measure questionnaires for both groups, whereas the post-intervention survey for both groups differed (Supplementary Materials; Questionnaires S1 and S2). The MTC group’s post-intervention survey included a detailed training course feasibility and acceptability questionnaire. In contrast, the WL group provided information about utilizing any kind of therapy or exposure to any form of mindfulness during the past eight weeks. Both assessments were completed through online survey links. The participants were sent reminders to complete assessment survey links, submit weekly worksheets and upcoming online sessions.

A clinical psychologist, unrelated to the research project, interviewed the consenting participants from the MTC group after the completion of the 8-week intervention. A semi-structured interview was conducted to assess the participants’ perceived acceptability of MTC and its different elements, barriers, and facilitators (Supplementary Materials; Document S1). The interviews lasted 20–25 min on average.

### 2.3. Mindfulness Training Course

The Mindfulness Training course was an 8-week course with group-based online weekly sessions with a course facilitator. It was an adaptation of the self-help book “Mindfulness: a practical guide to finding peace in a frantic world” [19], which incorporates the principles of both mindfulness-based stress reduction (MBSR) [39] and mindfulness-based cognitive therapy [40]. The book has been used as a guideline for developing mindfulness courses and programs for students focusing on stress reduction, wellbeing, resilience [2], and emotional regulation [18]. It has not only been found to be beneficial in different cultures [2,18], but has also been delivered as a self-help course to students [37], making it a worthy choice for an online mindfulness-based intervention.

The Mindfulness Training Course (MTC), translated and adapted in Urdu, included eight guided audio meditations and one additional audio recording. The additional audio recording was an overview and introduction of the forthcoming 8-week training (including orientation about what mindfulness is and an explanation of frequently used standard terms, the foundational attitude, and its importance in practice). MTC also included eight chapters corresponding to each week and worksheets to record home practice (formal and informal practices). Each week followed a theme: waking up to the autopilot, keeping the body in mind, the mouse in the maze, moving beyond the rumor mill, turning towards difficulties, practicing kindness, when did you stop dancing, and your wild and precious life. The formal practices included meditation practices guided through the audio tracks (breath and body, body scan, 3-minute breathing space, sounds and thoughts, mindful movement, exploring difficulties, and befriending meditation). In the “exploring difficulties meditation”, the participants were guided to use attitudes of curiosity and kindness to relate differently to thoughts, worries, problems, difficult situations, uncomfortable feelings, and sensations. In the “befriending meditation” they were guided into relating to themselves, the world, and difficulties with kindness, respect, and compassion. The informal practices included being mindful during everyday activities such as a “habit releaser”—process of loosening up habits by introducing a little randomness (e.g., deliberately changing chairs we usually sit in). The “raisin meditation” was practiced with the facilitator during the first session to awaken and explore all senses while eating a raisin/dry fruit/grape. Participants then continued this experience with their everyday meals, snacks and later on to everyday activities. Online group sessions with the facilitator were conducted weekly. The sessions included a 5-minute informal warm-up in which participants shared their thoughts (to facilitate the online mode of delivery) followed by a formal welcome, a recap of the previous week, and an introduction to the present week’s theme, which led into practicing the meditation for that week guided by the facilitator. After the meditation practice, the participants shared their experience, followed by a review of the previous week’s home practice. The session ended with setting home practice for the week and a poem befitting that week’s theme. After each session, the participants were emailed the required material for that week (weekly chapter, audio tracks, and home practice worksheets). The facilitator remained vigilant throughout the sessions, and if a participant appeared disturbed, the facilitator contacted them through email to see if they needed any help. The facilitator was available in the Zoom meeting 15 min before and after the group session, in case any participant needed to share anything. A description of each week’s theme with core exercises and home practice is provided in Table S1 (Supplementary Materials). The facilitator notes for each session can be made available on request.

The training was provided by AS, who completed the one-on-one 9-week MBSR teacher training course from https://home.mindfulness-network.org/ (accessed on 5 June 2019), an international certifying body for mindfulness teacher training. AS has five years of mindfulness practice experience under supervision.

### 2.4. Wait-List Control Group

The control group did not receive any intervention during the trial. They completed the baseline and post-assessments at the same time as the MTC group, following the same procedures. After completion of the project, they were offered the Mindfulness Training Course in accordance with ethical guidelines.

### 2.5. Measures

#### 2.5.1. Feasibility of Trial

The trial’s feasibility was measured quantitatively based on the number of participants recruited by the four recruitment methods, and qualitatively through the barriers and facilitators in each method reported by recruiters in their submitted report. It was further quantitatively assessed through the feasibility of videoconferencing (post-intervention survey for MTC group), eligibility criteria, randomization, online data collection procedures, and flexible multiple weekly online sessions (the number of participants making use of the option of choosing which group to attend each week).

#### 2.5.2. Acceptability of Trial

Students’ acceptance of videoconferencing for MTC was assessed (quantitatively and qualitatively) through a post-intervention survey with questions about ease of use, technical difficulties, overall satisfaction, and suggestions for improvement.

The trial’s acceptability was also assessed through attrition rate (the number of participants who did not complete outcome data) and consent to randomization. The reasons for refusal to be randomized were also recorded.

#### 2.5.3. Acceptability of Intervention

The intervention’s acceptability was assessed through the intervention dropout rate (the number of participants who actively informed the facilitator that they were withdrawing from the course and who stopped attending the course). The reasons for withdrawal and dropout were also recorded.

The acceptability of different intervention elements was further assessed through participant attendance in weekly online sessions while recording the reasons for missing sessions, the percentage of participants submitting home practice worksheets, and time spent in home practice (post-intervention survey).

The perceived acceptability of different elements of MTC, barriers, and facilitators to practice was assessed through a semi-structured interview conducted with consenting MTC group participants. The interview was conducted by a clinical psychologist not involved in the project.

### 2.6. Mental Health Outcome Measures

***Five-Facet Mindfulness Questionnaires-FFMQ*** [41]: FFMQ assesses different mindfulness aspects. It consists of 39 items, indicating five facets: non-reactivity to inner experience, observing, acting with awareness, describing and non-judging experience. Each item is rated on a 5-point Likert scale, with higher scores indicating an increase in dispositional mindfulness level. An Urdu translation of FFMQ was used [42] with reliability α = 0.81.

***Clinical Outcomes Routine Evaluation-Outcome Measure*-*CORE-OM*** [43]: CORE-OM is a 34-item scale used to assess overall psychological distress. In the present study, it is used as an indicator of stress reduction [2,43,44,45,46]. CORE-OM is a concise tool that covers three dimensions: subjective wellbeing, commonly experienced problems, and life functioning. In addition, it contains items on risk to self and others. The approved version of the Urdu translation of CORE-OM was used in this study with reliability α = 0.87. It was approved by the developer of CORE-OM and is available on their website [47].

***Ryff’s Psychological Wellbeing Scale-PWB-S*** Ryff [48]: Ryff’s PWB-S consists of six subscales that measure different domains of eudaimonic wellbeing. The 42-item version was used with subscales as autonomy, environmental mastery, personal growth, positive relations with others, purpose in life, and self-acceptance. The items are scored on a 6-point Likert scale. Higher scores indicate greater levels of psychological wellbeing. The Urdu version for this study, translated as part of the adaptation procedure, was used with reliability α = 0.88.

### 2.7. Data Analysis

This pilot trial employed methodological triangulation as each objective of the trial utilized quantitative and qualitative methods to analyze its respective elements. Both quantitative and qualitative data were obtained and analyzed separately and combined at the interpretation phase to obtain a more nuanced understanding of the results.

Trial feasibility outcomes were analyzed descriptively. Mental health outcomes (stress, wellbeing, and mindfulness) were analyzed using descriptive statistics, and means with standard deviation were reported. The mean differences between MTC and WL groups and the 95% confidence interval (CI) were estimated.

Trial acceptability outcomes were analyzed narratively. The narrative records for this outcome (including suggestions for improvement of videoconferencing mode) were analyzed with semi-structured interview transcripts.

Intervention acceptability outcomes were analyzed narratively. A detailed qualitative analysis was performed. The 18 semi-structured interviews conducted with participants, the session notes of the facilitator, and participant-filled worksheets were subjected to reflexive thematic analysis [49]. Both inductive and deductive approaches were used, in which the research questions guided the identification of the dataset, thus being deductive and theory driven, whereas once identified, the data extracts were open coded, data-driven, semantic, and inductive. The transcription and reflexive thematic analysis of the interviews was carried out manually by AS using Excel sheets to record iterations in themes and codes.

## 3. Results

The study results are presented following the guidelines for recommended practice in designing, analyzing, and reporting of pilot and feasibility studies [50]. Furthermore, we used Consolidated Standards for Reporting Trials (CONSORT) extension guidelines for pilot and feasibility studies [51,52]. The main aim of this study was to establish the feasibility and acceptability of conducting a randomized controlled trial for a culturally adapted Mindfulness Training course with online mode of delivery. In general, the results highlighted indicators for feasibility and acceptability of the MTC, as well as the trial procedures.

**Figure 1:**
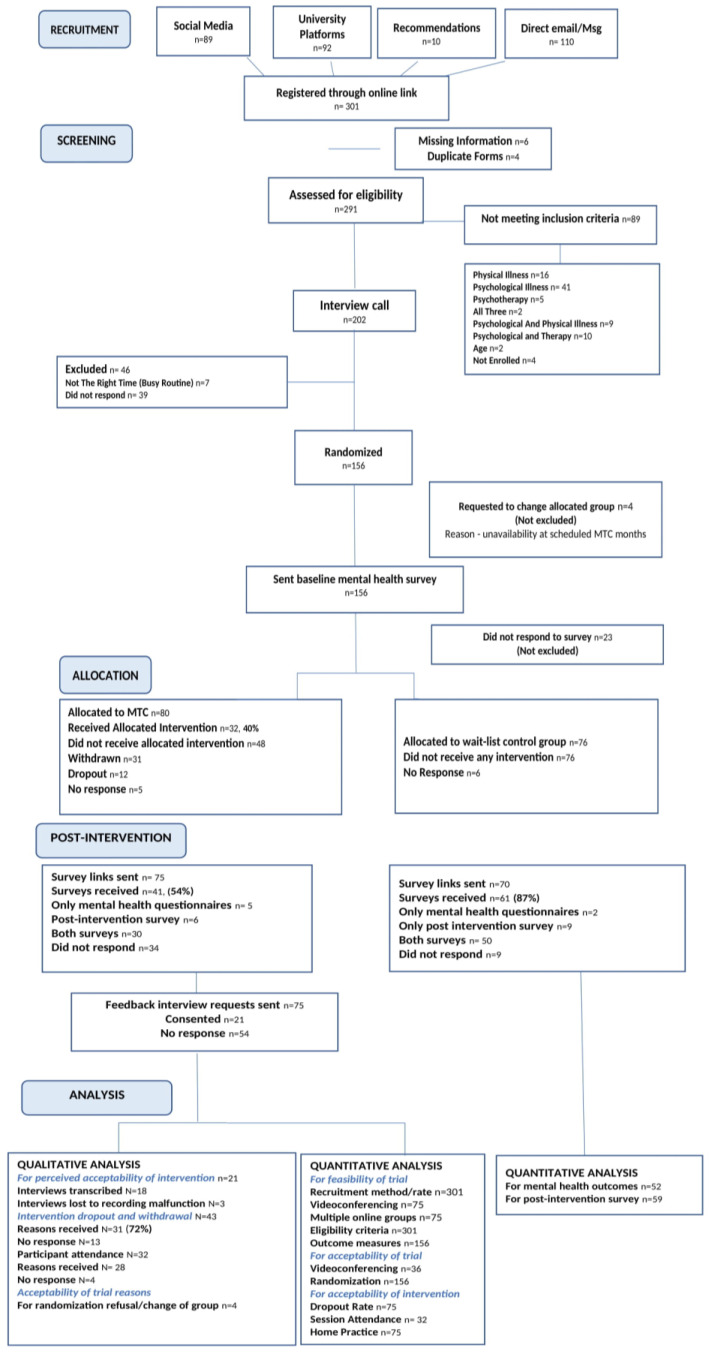
CONSORT Flow diagram for pilot randomized control trial of a Mindfulness Training Course for university students.

The CONSORT flow diagram (Figure 1) presents the flow of participants from recruitment until analysis, along with reasons where necessary.

At time of allocation, the two intervention groups were similar across demographics and baseline measures of mental health outcomes. The overall sample consisted of a greater number of females (*n* = 108, 69%) than males (*n* = 48, 30%); however, the MTC and WL groups did not have notable gender differences (Table 1). The factor which is most relevant to the present study is the similarity of the two groups in terms of their prior knowledge of mindfulness, with the majority of participants having either no knowledge (*n* = 57, 36%) or being new to mindfulness (*n* = 52, 33%), followed by those who had read about it (*n* = 36, 23%). There were more students from public universities (*n* = 134, 85%), than private universities (*n* = 22, 14.1%), which is representative of the student population in the public: private sector university ratio (80% in the public sector and 20% in the private sector; [53]). However, the MTC and WL groups were similar in the number of participants from public and private sector universities.

### 3.1. Trial Feasibility

Of the four *recruitment methods*, three methods were the most successful: direct contact with students via email or message resulted in the highest number of registrations (*n* = 110, 36%), followed by university platforms (*n* = 92, 30%) and social media (*n* = 89, 29%). Recommendations had the lowest number of registrations (*n* = 10, 3%) by the end of recruitment phase.

The RAs report on facilitators and barriers during the process highlighted *social media* as having greater outreach and easy dissemination, while it had the drawback of not addressing any of students’ initial queries, which might have led to their decision not to register for the course (Table 2). The recruitment method of *direct messaging* was reported by the RAs to have been facilitated by the students contacting them for information (especially through WhatsApp messages). According to the Ras, it had a personal, invested element where students, who are bombarded with tons of academia-related notifications, could respond as soon as they received the flyer. This method had the maximum number of registrations (36%). Flyers disseminated through University platforms (30%) were perceived by students as more credible; however, it had the pitfall of the study being seen as one of many courses being offered. The RAs did not report any barriers to the recommendation’s method, in which briefing about the expertise of the facilitator, the study, and institutional affiliation were seen as facilitating the process.

Analysis of the feasibility of *videoconferencing* for online sessions revealed that participants (MTC group, *n* = 75) faced certain problems that were categorized as connectivity issues (*n* = 9, 12%), technical difficulties such as electricity or device malfunctions (*n* = 1, 1%), or seeking extra assistance due to technical or connectivity issues (*n* = 4, 5%). These results show that connectivity and technical issues were faced by a small proportion of participants (13%), of which 5% overcame the issue by seeking extra assistance and the remaining participants reported having understood through material provided.

Further analysis of Zoom features based on facilitator notes showed that the chat function was used to share experiences and participate in activities in which audio was hindered due to connectivity issues. The hand-raising function was used to organize responses and ensure everyone had an opportunity to share. The thumbs up function was used to indicate an agreement or a yes response (finishing meditation, activity tasks, etc.). The heart function was used when participants resonated with a point. Thus, the participants creatively managed the connectivity issues using Zoom features and extra assistance (if deemed necessary). The video function was not used by all participants, as it hindered their connection; however, they were engaged from time to time to ensure their presence.

*Flexible weekly multiple online sessions* were conducted through three online groups per week (Saturday morning, Saturday evening, and Sunday morning). The same session was repeated in each Zoom meeting. This format seemed to facilitate the participants’ attendance and continuation with the course, as 42% of participants took advantage of this option and requested to change their allotted group based on their unavailability in the allotted time. In the reflexive thematic analysis of the interview transcripts, the code “multiple groups per session” was categorized as a MTC delivery related facilitator of participants’ mindfulness practice.

The *randomization process* went smoothly and as per protocol, with no difficulties encountered by the randomization researcher.

The *online data collection procedures* seemed feasible, with completion rates of more than 85% for the four assessments (Table 3). Participants were sent reminders for completion of baseline and post-intervention mental health outcome measures (16% and 12%, respectively), which might be due to their relatively greater average completion time (25 min and 22 min, respectively) compared to other two assessments.

### 3.2. Acceptability of Trial

The results from post-intervention survey (experimental group) quantifying the acceptability of videoconferencing (on a scale of 0–5, where 0 is “not at all” and 5 is “very much”), revealed that participants experienced considerable ease in using online videoconferencing (Mean = 4.43 (0.76 SD), Range = 3–5) and faced none to some technical difficulties during online sessions (M = 1.59 (0.64), R = 1–3) which included connectivity issues (37%) and voice fluctuations (10%). The overall satisfaction with online sessions was rated high (M = 4.32 (0.88), R = 2–5); the perceived effectiveness of online sessions was high (M = 4.43 (0.72), R = 3–5), with the understandability (M = 4.54 (0.69), R = 3–5) and relatability (M = 4.02 (0.76), R = 2–5) of the information provided during sessions rated towards the higher limits. The meditation exercises conducted during sessions were perceived to be effective (M = 4.35 (0.71), R = 3–5).

Regarding the *attrition rate* for this pilot trial, 34 of the 75 participants (45%) in MTC group did not respond to survey links sent post-intervention, despite reminders. Of the 41 surveys received (51%), 6 participants completed only the three mental health outcome questionnaires, 6 completed only the post-intervention survey, and 30 participants completed both the mental health questionnaires and the post-intervention survey. Interestingly, of the 45% participants who did not complete the surveys, 24 participants (32%) were those who dropped out or withdrew from the course and 10 participants (13%) were those who completed the course but did not complete the surveys. From the WL group, 9 of the 70 participants (12%) did not respond to the post-intervention survey links. Of the 61 received surveys (87%), 2 participants completed only the mental health questionnaires, 9 completed only the post-intervention survey, and 50 participants completed both surveys.

### 3.3. Acceptability of Intervention

Table 4 summarizes the participant’s attendance in weekly sessions, their reasons for missing sessions, the participants who submitted worksheets, the average time spent in meditations, daily participant engagement in mindfulness activities, and reasons for withdrawal and dropout from the MTC. Of the 80 participants allocated to MTC group, 48 (60%) did not receive the allocated intervention. Thirty-one participants withdrew from the course, informing the facilitator; twelve dropped out without informing the facilitator prior to leaving; and five participants did not respond after allocation. The forty-eight participants who dropped out, withdrew, or did not respond after allocation were not specific to any one recruitment method; hence, the method of recruitment did not seem to predict dropout. The 32 participants who completed the MTC attended 7 out of 8 sessions on average. Of the participants allocated to the MTC, 34% submitted weekly worksheets and on average, four worksheets out of eight were submitted by each participant. The average daily time spent in meditation by 54% participants was at least 10–15 min, whereas 48% engaged in daily mindfulness activities at least 1–2 times.

Table 5 summarizes the number of participants who submitted weekly worksheets and the number of participants who performed the respective weekly formal and informal practices based on their submitted weekly worksheets. The table shows the progressive decrease in the number of worksheets submitted across the weeks. Breath and body meditation was consistently continued throughout the course, except during week 2. The three-minute breathing space was carried out in all weeks, both formally and informally (breathing space). Mindful activity was also practiced informally across all weeks except the sixth week.

The interviews evaluating the perceived acceptability of different elements of the Mindfulness training course by the participants revealed that participants rated their overall satisfaction with the course as M = 8 on a scale of 0–10 (0 = least satisfied and 10 = extremely satisfied). The participants identified both formal and informal practices to be effective for them. Informally practiced breathing space, the ten-finger gratitude exercise, the kindness exercise, the grounding anchor, nourishing activities, habit releasers, and mindful walking were perceived as effective by participants because they were seen as being helpful for stress management and perspective taking, improved relational change, and helped with self-connection and coping with negative thoughts. Specific formal meditations that participants perceived to be helpful included breathing space, sounds and thoughts meditation, breath and body meditation, and mindful movement meditation. Participants’ time for practicing formal meditations was flexible and took place during walks, between classes, and before going to sleep. Participants deemed these meditations to be helpful in developing healthy activities, changing old habits, improving sleep quality, and recharging them for the day or the task ahead. The participants experienced interpersonal, intrapersonal, behavioral, and cognitive change along with improved wellbeing. The participants experienced a change in their relationships in different areas, including active listening, being accepting of others’ views, improved interactions, and expression, and becoming more compassionate and empathetic. Some participants experienced change in how they related to themselves through reduced self-criticism, increased self-connection, self-growth and taking more time for self-care within their busy lives. Some participants experienced a change in behavior in terms of reduced anger, improved professional skills of public speaking and work performance, stress management, and problem-solving skills. Some participants experienced improved memory and reduced ruminating and mind wandering. They also reported enrichened everyday experiences, efficient functioning, increased tolerance and gratitude, and the ability to shift perspectives.

An important theme deciphered from the transcripts was the mindfulness processes internalized by the participants. Most of the participants found themselves shifting from doing mode to being mode more often by becoming aware of their surroundings, thoughts, and emotions in the present moment. Some reported becoming more accepting and noticing their thoughts rather than engaging with them. The phenomenon of letting go was also reported by some participants.

The participants experienced certain facilitators and challenges during their mindfulness practice. The reported facilitators to mindfulness practice emerged in four themes: participant related, MTC delivery related, MTC elements related, and facilitator related. The aspects related to participants that they reported as facilitating their mindfulness practice included not being self-critical when they were unable to practice, seeing it as an experience, and adjusting their practices to “what works” instead of being rigid; their own motivation for self-development; setting reminders on their phone for daily practices and tasks; and continuing to attend sessions whether they had completed the weekly tasks or not. Some participants reported certain aspects of the way MTC was delivered to have facilitated their mindfulness practice. These included sessions held on weekends and multiple groups for each weekly session provided flexibility, ensuring their attendance even if they were unable to attend their initially assigned group. Further, the regular reminders sent for practices and sessions were seen as supporting their practice. Some participants found the group format supportive in encouraging them to practice and overcome their challenges through listening to others’ experiences and queries. Some participants reported specific elements of MTC to be helpful in their practice: the worksheets helped them reflect on their experiences and aided in gaining insight; home tasks made the course experiential; the readings helped understand and bring perspective to their understanding. Most of the participants found the sessions to be most helpful in their mindful practice. The supportive aspects of sessions reported by participants included the safe space of non-judgment, openness, and no pressure to speak, with acceptance during the sessions, the meditations practiced, and information provided during sessions and all queries being addressed with constructive feedback that helped them gain an understanding of their experience. Participants also reported the facilitator’s attitude and guidance as important factors supporting their mindful practice. The facilitator’s non-judgmental, accepting, and encouraging attitude, accommodating everyone’s needs with gentleness, was seen as supporting their practice, and it helped them practice these attitudes with themselves. Some participants found the facilitator’s insight into their experiences, guidance during their struggling moments, and availability after sessions to be supportive.

Participants reported resource-related and ability-related challenges that made mindfulness practice difficult for them. Most of the participants reported a hectic routine and academic work as posing a major challenge to their weekly task completions. Most of the participants found completing and submitting worksheets an overwhelming process, which felt like another task. A few participants found formal meditations challenging, as sitting still in silence with themselves was a new experience and felt uncomfortable, whereas a few reported finding a quiet place for meditation was a challenge due to family dynamics.

**Figure 2:**
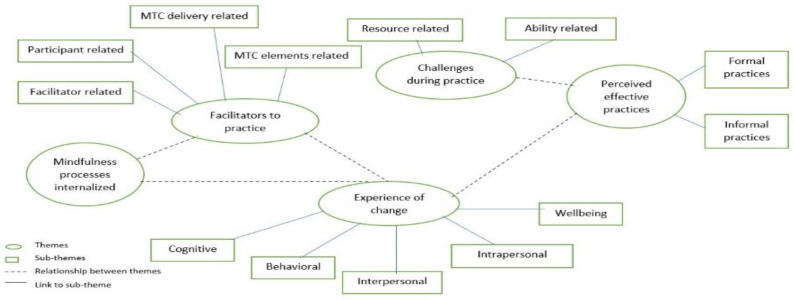
Thematic map demonstrating the five themes from reflexive thematic analysis of acceptability interviews.

The participants also provided specific suggestions and recommendations for future iterations of the course. Some suggested an audio recording of readings would be easier to manage with busy routines, whereas a few suggested decreasing the length of the weekly readings. Some participants suggested that this course is needed by all age groups and must be offered for all age groups including schools, that the outreach should be expanded so more youth can benefit from it, and that the course should be offered on a regular basis. Figure 2 illustrates the themes and subthemes, indicating the linkages between them. The themes with descriptions and quotes from participants are available in Table S2 (Supplementary Materials).

### 3.4. Mental Health Outcomes

Table 6 shows means, standard deviations, mean differences, and 95% confidence intervals across MTC and WLC groups at post intervention for the three mental health outcomes. The mean CORE-OM scores of MTC group were −28.2 (95% confidence interval −37.28 to −19.11) points lower than WL group. The MTC group mean scores on PWB-S and FFMQ were 11.75 (95%CI −0.12 to 23.63) and 12.5 (95% CI 3.93 to 21.06) points higher, respectively, compared to the WL group post-intervention.

A paired-sample t test was calculated to compare the scores obtained by the retained participants in the MTC group before and after the intervention (Table 7). There was a significant difference between pre- and post-assessment scores for FFMQ, PWB-S, and CORE-OM in the MTC group for the retained participants.

## 4. Discussion

The first aim of this study was to examine the feasibility of conducting a full-scale randomized controlled trial of an 8-week online Mindfulness training course for the wellbeing and stress of university students. The second aim was to establish the acceptability of this course for the participants. The study also aimed to assess preliminary effectiveness by examining the differences between the trial arms on mental health outcome measures. There were no prior statistical criteria identified for success of MTC, but in comparison with other similar studies conducted with university students, our results are promising. The identified areas for improvement are attrition and dropout rates.

The feasibility indicators of the pilot RCT show very encouraging results, with relatively high recruitment rates for three recruitment methods: *direct email/message* to students, *university platforms* and *social media*. The lowest number of registrations from the *recommendations* method could be indicative of the lowered outreach of recruiters to academics asking for recommendations. It could also be suggestive of academics’ knowledge of mindfulness and selective referral based on their understanding of certain students’ personal attributes and likelihood of completing the course. Overall, this method appeared to be more subjective and restricted compared to other methods. The high recruitment rate through directly approaching students and using university platforms have been the preferred methods in previous mindfulness research [15,16,17,18]. Social media is a new method which has not previously been used to recruit university students for Mindfulness courses, and a future definitive RCT could benefit from using these three methods together to increase outreach. Predominantly, the high enrollment of students for this trial in a 2-week period seems indicative of the greater need and acceptability of such platforms of mental health for this population. The sample of the present study was quite representative of the Pakistani university student body, showing that there is interest in this sort of intervention across the spectrum of students.

The results indicate the weekly online sessions are feasible and acceptable to students, who reported them as “facilitators to mindfulness practice”. In addition to this, there was a small proportion of students who faced technical issues; those who did managed by either taking extra guidance from the facilitator or going through the reading material. Earlier studies examining online mindfulness interventions for students have mostly used web-based or automated self-help online programs [37,46]. These studies did not include live interaction with a facilitator and the study by Cavanagh and colleagues [36] had a Western sample, which might also indicate culturally different educational practices between Pakistan and UK. The trial conducted by Mak and colleagues [46] sampled university students from China; however, being a website, it governed self-paced intervention, with a 74.7% attrition rate. The absence of human interaction cannot be ruled out as a possible factor. Since the study did not take feedback from the participants, the acceptability of this web-based course can be argued upon. To our knowledge, MTC is the first mindfulness course to feature weekly online interactive sessions with a facilitator and, based on the results, this mode of delivery can be cost effective, with greater outreach and accessibility for students in the definitive trial.

The results of all assessment surveys sent through online links indicate that this online data collection procedure is feasible. The results also indicate that reminders were needed only for mental health outcome surveys, which took more time compared to other surveys. Therefore, to reduce the time and reminders, brief versions for the outcomes (psychological wellbeing and distress) may be used in future trials.

The attrition rate for this study, which is seen as a perennial problem for online mindfulness interventions (74% [46]; 42% brief self-help MBI [37]), was relatively high in the MTC (45%),. Both these interventions were self-help mindfulness courses for university students with no sessions with a facilitator. The study by Cavanagh and colleagues [37], had a lower attrition rate in the Mindfulness arm, and it was a briefer version of MBI. The high attrition might be indicative of unmotivated individuals with certain personality attributes that did not make MBI a good fit for them. It is also possible that the participants did not find the materials and approach engaging enough. In our study, the drop out and withdrawal analyses showed that the major reason for leaving the course was quoted as having a “busy and hectic routine”. Meanwhile, the interviews with completers revealed that they also experienced “hectic routine” as a challenge during mindfulness practice, yet they somehow managed with whatever time they had available to stay engaged with the intervention. This might be explored in future studies to see if certain attributes, personality styles, motivations, or pre-existing skill sets are more conducive to cultivating mindfulness practice. It might be helpful to conduct orientation and pre-intervention sessions both online and in person at different universities to allow students to experience mindfulness before they choose to register. Hence, the high attrition rate does not seem to question the reliability and validity of the intervention itself; rather, it seems to be an attribute seen in student population in specific and online interventions in general [37], or due to lack of knowledge about the nature of mindfulness courses. This also points indirectly to the acceptability and feasibility of the online intervention modality. It has clear implications for further contextual adaptations that may be needed in future definitive trials or studies. While a much lower attrition rate of WL group is consistent with other study findings [37], it might need further exploration before initiating future larger trials, as mentioned above.

Regarding the attendance, course completion and home practice, the study results showed good compliance rates. Those who engaged with the MTC attended an average of 7/8 sessions and remained committed to practicing mindfulness throughout the course. This indicates that for those participants who engaged, the course was acceptable. However, the worksheet submission (34%) seemed to pose a problem, as indicated by the decrease in number of participants submitting home worksheets throughout the duration of the course. In the semi-structured interviews, worksheets submissions were reported to be both a challenge as well as supportive of participants’ self-understanding. The students have a busy routine, and filling worksheets seemed like another task to them; however, considering their beneficial role, it might help in future trials to include an incentive for submitting worksheets (e.g., money or gift vouchers). Breath and body meditation seemed to have been practiced consistently throughout the course, whereas 3-minute breathing space was practiced both formally and informally. Carrying out a mindful activity every day was also practiced regularly through the course. With reference to assessment completion, the participants who completed the course completed all surveys, whereas of the participants who withdrew or dropped out of the course, only 4 completed the surveys. This suggests that the assessments used in the study are acceptable to those who commit to the course. This phenomenon has been attributed as a reason for the high attrition rate in non-clinical population studies [10]. The baseline survey and post-assessment of mental health questionnaires required reminders (for a small number of participants) to be completed. In future studies, it might be more productive to use brief versions for the outcomes of stress and wellbeing. The randomization procedures were successful, as the baseline characteristics were similar in both groups and the majority of participants consented to being randomized. This suggests that randomization can be carried out in future studies.

The participants who engaged with the MTC reported being highly satisfied with the course, including the material and delivery. Both formal and informal mindfulness practices were perceived to have brought change within the participants, as well as in their relationships, work performance, and productivity. The participants benefited from the course when they practiced flexibly, tailoring it to their needs and work schedules. The internalization of mindfulness process was an important theme, which suggests the possibility of long-term effects of MTC. The facilitators and challenges identified by the participants seem to be connected, as they used mindfulness attitude of non-criticism, non-judgment, letting go, and being mode to manage the difficulties they faced in mindfulness practice, as well as their daily life. The multiple groups held for each weekly session and reminders sent for online sessions and tasks were seen as facilitating their commitment. In future studies, it would be helpful to continue with this format of multiple sessions and reminders to support engagement and practice. The facilitator’s attitude and guidance were seen as critical to participants’ mindfulness practice and understanding their experience, internalizing mindfulness attitude and processes. This finding is in line with studies emphasizing the role of a facilitator in the effectiveness of mindfulness courses [54,55]. In future studies, it would help to employ these findings from the Reflexive thematic analysis to develop models of mindfulness mechanisms, including the participant processes and the facilitator’s role with MTC elements, delivery, and mindfulness components. The suggestions of including audio reading can be incorporated into future studies. The recommendations for continuing the course, increasing its outreach to all ages and more students suggests the high acceptance and perceived effectiveness of MTC with university students. Regarding the quantitative results of mental health outcomes, it would be right to state that we did not aim to establish the effectiveness of MTC; however, because of over recruitment largely exceeding the intended sample size, the study may have become powered. FFMQ and CORE differences between groups were statistically significant. These results need to be confirmed in larger and purposively designed trials.

### Limitations

The study had a few limitations. One limitation of the present study was that the baseline questionnaires were filled after allocation, which might have biased the participants’ responses. Future trials could address this by allocating participants to trial arms after baseline questionnaires are filled. Second, self-report as assessment of eligibility criteria could be complemented by using screening tools with cut-off scores to establish mental health problems. Third, outcome self-reporting in open label trials (i.e., those in which participants know their allocation) is subject to reporting bias. However, blinding participants in this case would be extremely hard due to the behavioral nature of the intervention. Fourth, future trials could benefit from 3–6 month follow-up assessment of qualitative themes, as well as mental health outcome measures. Finally, statistical analyses were of a very preliminary nature and need confirmation via a definitive trial complemented by an in-depth content analysis.

## 5. Conclusions

In summary, this was a randomized controlled trial with two branches attempting to examine the feasibility and acceptability concerns surrounding the delivery of Mindfulness Training Course for university students in Pakistan. The study highlighted important methodological and process steps for future definitive RCTs regarding recruitment, randomization, online data collection, attrition, and drop out. Our results show that for the participants who completed the intervention (*n* = 32), MTC was acceptable and in a short period of time (2 weeks), many students registered, which can be increased significantly in a large-scale RCT. The participant engagement and adherence were encouraging. Although the retention was lower in relation to the eligible participants randomized, the feedback from participants was generally positive and encouraging. The trial also provided specific details about the intervention delivery, highlighting the acceptability of elements of MTC, including multiple weekly online sessions, reminders, home practice, the role of the facilitator, and formal and informal practices.

It would be helpful to increase the outreach to universities and students across the country in future definitive trials, initiating the recruitment process through orientation and taster sessions to improve retention. The worksheet submission aspect can be improved by offering incentives of some kind for each weekly submission. Finally, in any future randomized trial, a follow-up of 3–6 months after the post-intervention assessment could be insightful in understanding the qualitative themes of “mindfulness processes internalized” and the benefits of formal and informal practices.

In relation to the wider context of Pakistani students’ added challenges of political instability, socio-economic struggles, lowered access to professional help [26], and attitude towards mental health [27,28], MBIs might facilitate their resilience and functioning to some extent; however, they cannot possibly address all of these factors. Social action is needed to address structural issues to improve mental health further.

## Figures and Tables

**Table 1:** Baseline information of the randomized participants.

Parameter	Total (*n* = 156)	MTC Group (*n* = 80)	WL Control Group (*n* = 76)	Group Difference
**Age in years M (SD)**	22.90 (3.57)	23.08 (3.93)	22.72 (3.16)	0.36
**Gender M (%)**				
Male	48 (30.8%)	27	21	6
Female	108 (69.2%)	53	55	2
**Degree enrolled M (%)**				
Bachelors	79 (50.6%)	45	34	11
Masters	74 (47.5%)	32	42	10
Doctorate	3 (1.9%)	3	0	3
**Subject Major M (%)**				
Social Sciences	113 (72.4%)	57	56	1
Natural Sciences	14 (9.0%)	8	6	2
Engineering	29 (18.6%)	15	14	1
**Prior knowledge of Mindfulness M (%)**				
No knowledge	57 (36.5%)	29	28	1
New to Mindfulness	52 (33.3%)	26	26	0
Read about it	36 (23.8%)	19	17	2
Some experience	10 (6.4%)	5	5	0
**University M (%)**				
Private	22 (14.1%)	12	10	2
Public	134 (85.9%)	68	66	2
**Mental health measures M (SD)**	-	***n* = 74**	***n* = 73**	
FFMQ	-	116.64 (21.3)	117.61 (18.2)	0.97
PWB-S	-	169.43 (34.8)	175.72 (26.48)	6.29
CORE-OM	-	68.05 (14.6)	66.0 (13.1)	2.05

MTC = Mindfulness Training Course, WL = Wait-list control group, FFMQ = Five Facet Mindfulness Questionnaire, PWB-S = Psychological wellbeing Scale, CORE-OM = Clinical Outcomes Routine Evaluations–Outcome Measure.

**Table 2:** Barriers and Facilitators to recruitment methods.

Recruitment Methods	Barriers to Recruitment	Facilitators to Recruitment
Social media and student groups	Students’ lack of knowledge about mindfulness Being lost in news feed	Greater outreach
University platforms	Students’ perception of this being just another course	Perceived as credible, being advertised on an official platform
Recommendations	-	Briefing about the expertise of the facilitator and institutional affiliation Briefing about the study
Direct message/email	Being lost in email/spam	Contacting the recruiter for more information via message

**Table 3:** Online data collection of all assessments with completion rate, completion time, and reminders for completion.

Assessments	Completion Rate (%)	Average Completion Time (Minutes)	Participants Sent Reminders N (%)
Baseline Assessment of mental health outcomes	86	25	22 (16)
Post-intervention survey—MTC group	100	12	0
Post-intervention survey—WL control group	100	5	0
Post intervention—mental health outcomes	88	22	13 (12)

**Table 4:** Summary of participant engagement with MTC elements: sessions, worksheets, meditations, and mindful activities.

Indicators	Participant Responses
Reasons for missing sessions (N = 75)	Connectivity (12%)
	Academic commitments (36%)
	Health issues (12%)
	Travelling (15%)
	Family commitments (25%)
Percentage of participants submitting worksheets (N = 75)	34%
Time spent in meditation on average (N = 37)	0 min (8%)
	5 min (24%)
	10–15 min (54%)
	20–25 min (14%)
No. of times engaged in mindfulness activities/day (N = 37)	1–2 times (48%)
	3–4 times (40%)
	>5 times (12%)
Reasons for withdrawal and dropout (N = 43)	Connectivity (6%)
	Health issue (25%)
	Busy (39%)
	No reason (30%)

**Table 5:** Weekly Formal and informal mindfulness practices completed by participants (weekly submitted worksheets).

Weeks	1	2	3	4	5	6	7
	*n* = 26	*n* = 16	*n* = 15	*n* = 9	*n* = 10	*n* = 10	*n* = 9
**Formal Practices**							
Breath and body	16	-	7	5	3	5	2
Body scan	-	11	-	-	-	-	1
3-minute breathing space	-	-	8	5	4	3	5
Mindful movement	-	-	7	-	-	-	2
Sounds and thoughts	-	-	-	8	6	-	2
Exploring difficulties	-	-	-	-	9	-	2
Befriending	-	-	-	-	-	8	3
**Informal practices**							
Raisin meditation	6	-	-	-	-	-	
Habit releasers	7	3	1	-	-	-	
Mindful activity	26	11	8	1	2	-	3
Ten-finger gratitude exercise	-	7	-	-	-	-	1
Act of kindness	-	-	-	-	-	6	1
Nourishing activities	-	-	-	-	-	-	3
Breathing space	-	-	8	3	2	5	5

The shaded boxes indicate that the practice was not introduced in these weeks.

**Table 6:** Comparison of mental health outcome measures at post intervention.

Mental Health Outcome Measures	MTC N = 36 (M, SD)	WL N = 52 (M, SD)	Mean Difference (*p* Value)	95%CI
Psychological wellbeing (PWB-S)	179.38 (30.37)	167.63 (27.14)	11.75 (0.05)	−0.12 to 23.63
Mindfulness (FFMQ)	131.33 (21.)	118.83 (19.81)	12.5 (0.005)	3.93 to 21.06
Distress (CORE-OM)	33.16 (22.68)	61.36 (21.09)	−28.2 (0.000)	−37.28 to −19.11

MTC = Mindfulness Training Course, WL = Wait-list group, PWB-S = Psychological wellbeing scale, FFMQ = Five Facet Mindfulness Questionnaire, CORE-OM = Clinical outcomes routine evaluation-outcome measure. M = mean, CI = Confidence interval, SD = Standard deviation.

**Table 7:** Comparison of MTC group mental health outcome measures at pre- and post-intervention assessment (N = 36).

Mental Health Outcome Measures	Mean Pre–Post-Difference	SD	t	*p*	CI
Psychological wellbeing (PWB-S)	−9.80	24.60	−2.39	0.02	−18.13–−1.48
Mindfulness (FFMQ)	−17.97	22.18	−4.86	0.000	−25.47–−10.46
Distress (CORE-OM)	36.00	19.92	10.83	0.000	29.25–42.74

MTC = Mindfulness Training Course, PWB-S = Psychological wellbeing scale, FFMQ = Five Facet Mindfulness Questionnaire, CORE-OM = Clinical outcomes routine evaluation-outcome measure, CI = Confidence interval, SD = Standard deviation.

## Data Availability

The raw datasets analyzed during this study are available from the corresponding author on request.

## Supplementary Materials

The following supporting information can be downloaded at: https://www.mdpi.com/article/10.3390/ijerph20085512/s1. The manuscript refers to the following supplementary material available in the supplementary downloadable section. Table S1: Description of 8-week Mindfulness Training Course. Table S2: Themes, subthemes and sample quotes from acceptability interviews. Questionnaire S1: Post-intervention Survey–Waitlist group. Questionnaire S2: Post-intervention Survey-MTC group. Document S1: Semi-structured interview guide to explore the acceptability of students for MTC.

## References

[1] Dankaert E.S. (2013). The Relationship between Mindfulness and Eudaimonic Well-Being in South African University Students. Ph.D. Thesis.

[2] Galante J., Géraldine D., Maris V., Adam P., Wagner J.S., Alice B., Neal L., Emma H., Peter B.J. (2018). A mindfulness-based intervention to increase resilience to stress in university students (the Mindful Student Study): A pragmatic randomised controlled trial. Lancet Public Health.

[3] Nyklíček I., Karlijn F.K. (2008). Effects of mindfulness-based stress reduction intervention on psychological well-being and quality of life: Is increased mindfulness indeed the mechanism?. Ann. Behav. Med..

[4] Bóo S.J.M., Jasmine C.-F., Steven C., Bella D., Géraldine D., Peter B.J., Julieta G. (2020). A follow-up study to a randomised control trial to investigate the perceived impact of mindfulness on academic performance in university students. Couns. Psychother. Res..

[5] Miralles-Armenteros S., Ricardo C.-G., Alma R.-S., Zina B. (2021). Mindfulness and academic performance: The role of compassion and engagement. Innov. Educ. Teach. Int..

[6] Powietrzynska M., Kenneth T., Konstantinos A. (2015). Facing the grand challenges through heuristics and mindfulness. Cult. Stud. Sci. Educ..

[7] Gouda S., Minh T., Luong S.S., Joachim B. (2016). Students and teachers benefit from mindfulness-based stress reduction in a school-embedded pilot study. Front. Psychol..

[8] Kabat-Zinn J. (2003). Mindfulness-Based Interventions in Context: Past, Present, and Future.

[9] O’Driscoll M., Stephen B., Helen B., Sharon L., Laura J.S. (2019). An online mindfulness-based intervention for undergraduate pharmacy students: Results of a mixed-methods feasibility study. Curr. Pharm. Teach. Learn..

[10] Dawson A.F., William W., Brown J.A., Bella D., James N., Donald K.H., Sophie A., Tom B.M., Peter B.J., Julieta G. (2020). Mindfulness-based interventions for university students: A systematic review and meta-analysis of randomised controlled trials. Appl. Psychol. Health Well-Being.

[11] Wu H., Esther G., Norma G. (2015). International student’s challenge and adjustment to college. Educ. Res. Int..

[12] Dimidjian S., Zindel V.S. (2015). Prospects for a clinical science of mindfulness-based intervention. Am. Psychol..

[13] Michalak J., Johannes M., Thomas H. (2020). Implementation and dissemination of mindfulness-based interventions. Mindfulness.

[14] Ajilchi B., Hamid R.A., Zahra P.A., Majid M.Z., Steve K. (2019). Applying mindfulness training to enhance the mental toughness and emotional intelligence of amateur basketball players. Australas. Psychiatry.

[15] Demarzo M.M.P., Solange A., Nadia S., Sergio P., Sandra F., Javier G.-C. (2014). Mindfulness-based stress reduction (MBSR) in perceived stress and quality of life: An open, uncontrolled study in a Brazilian healthy sample. Explor. J. Sci. Heal..

[16] dos Santos T.M., Elisa H.K., Isabel S.C., Luiza H.T., Shirley S.L., Luiz A.N.-M. (2016). Positive effects of a stress reduction program based on mindfulness meditation in Brazilian nursing professionals: Qualitative and quantitative evaluation. Explore.

[17] Damião N.A., Alessandra L.G.L., Oscarina D.S.E., Giancarlo L. (2020). Effects of a required large-group mindfulness meditation course on first-year medical students’ mental health and quality of life: A randomized controlled trial. J. Gen. Intern. Med..

[18] Chiodelli R., Luana T.N.M., Saul N.J. (2018). Effects of a brief mindfulness-based intervention on emotional regulation and levels of mindfulness in senior students. Psicol. Reflexão E Crítica.

[19] Williams M., Danny P. (2011). Mindfulness: A Practical Guide to Finding Peace in a Frantic World.

[20] Phang K.C., Mukhtar F., Ibrahim N., Ling S.K., Mohd M.S. (2015). Effects of a DVD-delivered mindfulness-based intervention for stress reduction in medical students: A randomized controlled study. Educ. Med. J..

[21] Cavanagh K., Clara S., Lewis F., Fergal J. (2014). Can mindfulness and acceptance be learnt by self-help?: A systematic review and meta-analysis of mindfulness and acceptance-based self-help interventions. Clin. Psychol. Rev..

[22] González-García M., Jorge C.Á., Elena Z.P., Samuel F.-C., Javier G.L. (2021). Feasibility of a brief online mindfulness and compassion-based intervention to promote mental health among university students during the COVID-19 pandemic. Mindfulness.

[23] Khan M.M. (2016). Economic burden of mental illnesses in Pakistan. J. Ment. Health Policy Econ..

[24] Khan M.S., Sajid M., Areef B., Syed U.A., Yasir J. (2006). Prevalence of depression, anxiety and their associated factors among medical students in Karachi, Pakistan. J.-Pak. Med. Assoc..

[25] Syed A., Syed S.A., Muhammad K. (2018). Frequency of depression, anxiety and stress among the undergraduate physiotherapy students. Pak. J. Med. Sci..

[26] Sharan P., Gallo C., Gureje O., Lamberte E., Mari J.J., Mazzotti G., Patel V., Swartz L., Olifson O., Levav I. (2009). Mental health research priorities in low-and middle-income countries of Africa, Asia, Latin America and the Caribbean. Br. J. Psychiatry.

[27] Marcus M. (2009). What are young adults saying about mental health? A qualitative analysis of Internet Blogs. Medicine 2.0 Conference.

[28] Waqas A., Muhammad Z., Hamzah G., Muhammad W.U., Muhammad Z.T. (2014). Public stigma associated with mental illnesses in Pakistani university students: A cross sectional survey. PeerJ.

[29] Crane R.S., Judson B., Christina F., Jon K.-Z., Saki S.J., Mark G.W., Willem K. (2017). What defines mindfulness-based programs? The warp and the weft. Psychol. Med..

[30] Skivington K., Lynsay M., Sharon A.S., Peter C., Janis B., Jane M.B., Kathleen A.B., Neil C., David P.F., Emma M. (2021). A new framework for developing and evaluating complex interventions: Update of Medical Research Council guidance. BMJ.

[31] Barrera M., Felipe G.C. (2006). A Heuristic Framework for the Cultural Adaptation of Interventions.

[32] Sarfraz A., Siddiqui S. (2023). Cultural Adaptation of a Mindfulness-Based Intervention for Young Adults: An Application of Heuristic Framework. Pak. J. Soc. Res..

[33] Sarfraz A., Salma S. Addressing Students’ Stress in Higher Education through an Online Mindfulness Training Course. Proceedings of the 33rd BARCELONA International Conference on “Education, Humanities, Social Sciences & Arts” (EHSSA-22).

[34] Lewis M., Kieran B., Christopher J., Sutton G.M., Helen L.M., Gillian A.L. (2021). Determining sample size for progression criteria for pragmatic pilot RCTs: The hypothesis test strikes back!. Pilot Feasibility Stud..

[35] Julious S.A. (2005). Sample size of 12 per group rule of thumb for a pilot study. Pharm. Stat. J. Appl. Stat. Pharm. Ind..

[36] Alsubaie M., Chris D., Barnaby D., Dunn A.G., Obioha C., Ukoumunne A.E., Rachael V., Manish G., Willem K. (2020). Feasibility and acceptability of mindfulness-based cognitive therapy compared with mindfulness-based stress reduction and treatment as usual in people with depression and cardiovascular disorders: A three-arm randomised controlled trial. Mindfulness.

[37] Cavanagh K., Alasdair C., Puffin O.H., Thomas M., Phoebe V., Fergal J., Jenny G., Clara S. (2018). A randomised controlled trial of a brief online mindfulness-based intervention in a non-clinical population: Replication and extension. Mindfulness.

[38] Spijkerman M.P.J., Wendy T.M.P., Bohlmeijer E. (2016). Effectiveness of online mindfulness-based interventions in improving mental health: A review and meta-analysis of randomised controlled trials. Clin. Psychol. Rev..

[39] Kabat-Zinn J. (1982). An outpatient program in behavioral medicine for chronic pain patients based on the practice of mindfulness meditation: Theoretical considerations and preliminary results. Gen. Hosp. Psychiatry.

[40] Segal Z.V., John D.T., Williams M.J.G. (2004). Mindfulness-Based Cognitive Therapy: Theoretical Rationale and Empirical Status. Mindfulness and Acceptance: Expanding the Cognitive-Behavioral Tradition.

[41] Baer R.A., Smith G.T., Hopkins J., Krietemeyer J., Toney L. (2006). Five facet mindfulness questionnaire. Assessment.

[42] Niazi S., Adnan A. (2017). Role of mindfulness and psychological wellbeing between external locus of control and depression: A moderated mediation model. Peshawar J. Psychol. Behav. Sci. (PJPBS).

[43] Evans C., Janice C., Michael B., Frank M., Graeme M., John M.-C., Kerry A. (2002). Towards a standardised brief outcome measure: Psychometric properties and utility of the CORE–OM. Br. J. Psychiatry.

[44] Kang Y.S., So Y.C., Eunjung R. (2009). The effectiveness of a stress coping program based on mindfulness meditation on the stress, anxiety, and depression experienced by nursing students in Korea. Nurse Educ. Today.

[45] Phang C.K., Firdaus M., Normala I., Shian-Ling K., Sherina M.S. (2015). Effects of a brief mindfulness-based intervention program for stress management among medical students: The Mindful-Gym randomized controlled study. Adv. Health Sci. Educ..

[46] Mak W.W.S., Amy T.Y.C., Eliza Y.L.C., Cherry L.Y.L., Karin C.S.N. (2015). Enhancing Web-based mindfulness training for mental health promotion with the health action process approach: Randomized controlled trial. J. Med. Internet Res..

[47] Core Systems Trust Approved CORE Translations. https://www.coresystemtrust.org.uk/home/translations/urdu/.

[48] Ryff C.D. (1989). Happiness is everything, or is it? Explorations on the meaning of psychological well-being. J. Personal. Soc. Psychol..

[49] Braun V., Victoria C. (2019). Reflecting on reflexive thematic analysis. Qual. Res. Sport Exerc. Health.

[50] Thabane L., Gillian L. (2019). A guide to the reporting of protocols of pilot and feasibility trials. Pilot Feasibility Stud..

[51] Lancaster G.A. (2015). Pilot and feasibility studies come of age!. Pilot Feasibility Stud..

[52] Eldridge S.M., Claire L., Chan M.J., Campbell C.M., Bond S.H., Lehana T., Gillian A.L. (2016). CONSORT 2010 statement: Extension to randomised pilot and feasibility trials. BMJ.

[53] Dawood S., Khawaja S.H., Muhammad I.K., Muhammad Y., Muhammad B.K., Zubair F.P., Muhammad A.Z., Muhammad I.J. (2020). National Education Management Information System, Academy of Educational Planning & management with Technical and Financial Support from UNICEF, Pakistan: Pakistan Education Statistics 2017–2018.

[54] Bamber M.D., Joanne K.S. (2016). Mindfulness-based meditation to decrease stress and anxiety in college students: A narrative synthesis of the research. Educ. Res. Rev..

[55] Alampay L.P., Lourdes J.T., Galvez T., Antover P., Tuliao P.B., Mira A.O., Gilda D.L., Karina G.F., Patricia R., Angelique V. (2020). A pilot randomized controlled trial of a mindfulness program for Filipino children. Mindfulness.

