# Supplementary material for: Feasibility and Acceptability of an Online Mindfulness-Based Intervention for Stress Reduction and Psychological Wellbeing of University Students in Pakistan: A Pilot Randomized Controlled Trial
Source: Int J Environ Res Public Health. 2023 Apr 14;20(8):5512. doi: 10.3390/ijerph20085512 (PMC10139103; doi:10.3390/ijerph20085512)
Supplement: Supplementary file 1 [file ijerph-20-05512-s001.zip › ijerph-2300506-supplementary.pdf]

Supplementary material for the manuscript titled: Feasibility and Acceptability of an Online Mindfulness-Based Intervention for Stress Reduction and Psychological Wellbeing of University Students in Pakistan: A Pilot Randomized Controlled Trial

Table S1: Description of 8-week Mindfulness Training Course.

Weeks	The theme of the week	Core exercises and home practice for the week	Audio tracks for the week
**Week 1**	Waking up to the "autopilot"-building a capacity for sustained mindful concentration and awareness.	1. Raisin meditation-awakening and exploring all senses to be aware of an object<br>2. Mindful awareness of routine daily activity (e.g. drinking tea, taking a shower)<br>3. Mindfulness of Body and Breath meditation<br>4. Habit releaser-process of loosening up habits by introducing a little randomness (e.g deliberately changing chairs we usually sit in)<br>5. Practice eating at least one snack (fruit, sandwich) mindfully-with awareness of all senses and notice how it feels.	• Audio track 1-guided meditation for mindfulness of body and breath<br>• Audio track 9-introduction to the Mindfulness Training
**Week 2**	Keeping the body in mind-see the mind's reactivity by learning to pay mindful attention to the body and reintegrating mind and body into a powerful and seamless whole.	1. Body scan practice-practice being aware of different parts of the body<br>2. Carrying out another routine activity *mindfully* - a different one from last week (e.g drinking tea)<br>3. Ten finger gratitude exercise<br>4. Appreciation of here and now-mindfulness (being aware of thoughts, feelings, emotions, body sensations and action impulses) during pleasant events.<br>5. Habit releaser-going for a walk	• Audio track 2-guided meditation for body scan practice
**Week 3**	The mouse in the maze-mindfulness in daily life	1. Twelve minutes of Mindful Movement meditation -stretching exercise to realign body and release stress building up in the day.<br>2. Eight-minute Breath and Body meditation<br>3. A three-Minute Breathing Space meditation- concentrating core elements of the program in 3 steps for roughly 1 minute each to maintain compassionate, mindful stance and dissolve negative thought patterns before they gain control. It's an emergency meditation allowing us to see clearly what is arising from moment to moment when we feel under pressure.	• Audio track 3-gudied meditation for Mindful movement.<br>• Audio track 4-guided Breath and body meditation<br>• Audio track 8-guided meditation for three-minute Breathing space

		4. Habit Releaser-valuing the television	
**Week 4**	Moving beyond the rumor mill- relating differently to thoughts and worries	1. An eight-minute Breath and Body meditation<br>2. An eight-minute Sounds and Thoughts meditation.<br>3. A three-Minute Breathing Space meditation.	• Audio track 4- guided Breath and body meditation<br>• Audio track 5- guided sounds and thoughts meditation.<br>• Audio track 8- guided meditation for three-minute Breathing space
**Week 5**	Turning towards difficulties-from reacting to responding and moving towards acceptance	1. Eight-minute Breath and Body meditation<br>2. Eight-minute Sounds and thoughts meditation-<br>3. Ten-minute exploring Difficulty meditation-awareness towards how the body reacts during unsettling situations and cultivating the capacity to respond.<br>4. Breathing Space meditation.<br>5. Reviewing an unpleasant event-(awareness of thoughts, emotions, body sensations and action impulses) during the event.<br>6. Habit Releaser	• Audio track 4- guided Breath and body meditation<br>• Audio track 5- guided sounds and thoughts meditation<br>• Audio track 6- guided Exploring difficulty meditation<br>• Audio track 8- guided meditation for three-minute Breathing space
**Week 6**	Trapped in the past or living in the present- Practicing Kindness-	1. Ten-minute Befriending meditation<br>2. To prepare for befriending meditation, Breath and body meditation or Body and breath meditation (optional)<br>3. Breathing Space meditation.<br>4. Scenario exercise- Recognizing and disengaging from unhelpful self-critical habits<br>5. Habit releaser- random act of kindness or reclaiming your life (choosing any activity that brought joy and contentment before life became frantic or which was stopped being done due to busy life)	• Audio track 7- guided Befriending meditation<br>• Audio track 1- guided meditation for mindfulness of body and breath<br>• Audio track 4- guided Breath and body meditation<br>• Audio track 8- guided meditation for three-minute Breathing space
**Week 7**	When did you stop dancing? Reflecting on choices made in daily life and rebalancing the nourishing and depleting activities.	1. Choosing a formal meditation from – audio tracks that you find nourishing, Silent mindfulness of body and breath practice,sitting meditation.<br>2. Breathing space + action step- after practicing the 3 minute breathing space, carrying out a deliberate action cultivated through awareness.<br>3. Rebalancing exercise- adjusting the ratio of nourishing and depleting activities by becoming aware of the mode of mind.	Audio track 1-guided meditation for mindfulness of body and breath<br>Audio track 2- guided meditation for body scan practice<br>Audio track 3-gudied meditation for Mindful movement.<br>Audio track 4- guided Breath and body meditation<br>Audio track 5-guided sounds and thoughts meditation

		4. Doing at least two nourishing activities	Audio track 6- guided Exploring difficulty meditation<br>Audio track 7- guided Befriending meditation<br>Audio track 8- guided meditation for three-minute Breathing space
**Week 8**	Your wild and precious life- weaving your own parachute – using mindfulness to maintain your peace in a frantic world	1. Summing up the previous week themes and exercises<br>2. Identifying the themes most important to you.<br>3. Make a list of things you will practice after the end of this training (e.g starting day with breathing space, maintain formal practice, practice recognizing and befriending your feelings, taking a breathing space when feeling unsettled, bring mindfulness to daily activities, increasing level of exercise, remembering the breath and grounding attention in lower half of the body).<br>4. Setting the intentions for practice during next 8 weeks, by writing them down.	Audio track 1-guided meditation for mindfulness of body and breath<br>Audio track 2- guided meditation for body scan practice<br>Audio track 3-gudied meditation for Mindful movement.<br>Audio track 4- guided Breath and body meditation<br>Audio track 5-guided sounds and thoughts meditation<br>Audio track 6- guided Exploring difficulty meditation<br>Audio track 7- guided Befriending meditation<br>Audio track 8- guided meditation for three-minute Breathing space

Table S2: Themes, subthemes and sample quotes from acceptability interviews

Themes and subthemes	Summary	Quotes
1.Experienced change<br>1.1. Intrapersonal<br>1.1.1 Relation to self<br>1.2 Behavioral change<br>1.3 Cognitive change<br>1.4 Improved well-being<br>1.5. Interpersonal	The participants experienced change in themselves and in their relationships. They experienced reduced criticism, increased connection, growth, and care in relation to themselves. Participants noticed improved well-being in terms of enriched experiences, increased tolerance, gratitude with a change in perspective and efficient functioning. They noticed reduced anger and improved stress management, professional skills, problem solving, confidence. A few also reported increased attendance and improved sleep. Participants further experienced reduced ruminations and mind-wandering with improved memory. They observed improved interactions, increased expressiveness and patience in their relationships. Some participants also noticed practicing compassion, empathy and active listening and accepting of others' views during communications	"I used to get distracted, and my mind would go here and there while talking, sometimes in the past or in another thought, but now I have learned to remain in the present and calm myself down, yes this is the major change."<br>"I used to stay confused all the time, didn't feel like doing anything, felt lazy. So, after this course that laziness has gone. Not going to university, not feeling pleasure in anything, that all has finished now."<br>"First, I used to stop and stutter during presentations, but now I was able to concentrate, and it went smoothly. Others also noticed this."<br>"The activities of focusing in the present are a real privilege in our times as we get too work oriented in this fast paced life and forget ourselves. I learned how to incorporate things in my daily life for my own self."<br>"I experienced personal level change after the course. Like once I felt anger rising, but I noticed it arising and did not burst out in anger at that time."
2. Mindfulness processes internalized	The participants reported mindfulness processes becoming a part of them even after the course and when they were not engaging in mindful practices. They reported becoming aware of thoughts, acknowledging them, accepting and letting go with non-reactivity. The noticed shifting from doing mode into being mode.	"If I honestly tell, I didn't know there was any thing as being mode and doing mode……Now I become aware of what I'm doing and how I 'm doing it and how to adjust myself in the situation and enjoy the moment. Previously I didn't know how to enjoy."<br>"I have noticed that since this course started I have stopped criticizing myself for anything wrong, as what's done is done. Like during class when someone says something which is embarrassing, previously I would spend the whole week thinking about it, putting myself down for it,

		but now I don't do that, instead I let it go."<br>"It happened that I put my alarm to wake up in the night to study, but I woke up in the morning , because of this course, instead of getting into my negative thoughts that nothing can be done now and I'll fail, I took a pause, sorted my thoughts that criticizing myself will not help me, and I became calmer and ready for exam." "During exam, due to lack of sleep it so happened that I got blank and though I knew the answer I couldn't get what it was, so instead of criticizing myself, I took a pause, and then opened my eyes and I remembered the answer."<br>"This is how stress reduces mainly, that yes I am noticing the thoughts, that yes this thought is here, ok! but I am not letting them take over me, I am not engaging with them."
3. Perceived effective practices<br>3.1 Formal<br>3.1.1 specific meditations<br>3.1.2 benefits<br>3.1.3 Time of practice<br>3.2 Informal<br>3.2.1specific practices<br>3.2.2 benefits	Both formal and informal practices were perceived as effective by participants. In informal practices breathing space, ten finger gratitude exercise, habit releasers, kindness exercise, mindful walking, grounding anchor and nourishing activities were seen to benefit in stress management, perspective taking, self-connection, relational change and coping with negative thoughts. In formal practices mindful movement, breath and body meditation, befriending meditation and sounds and thoughts meditation were practiced before sleep, during walk or between classes. They helped in developing healthy activities, changing old habits, recharging energy and sleep quality.	"The meditation in the start, the breath and body meditation, where we focus on specific parts of body and breath, well…. I used to do it before going to bed and my sleep became peaceful and I didn't wake up during the night."<br>"So when I had 4 classes in a row, and a little break, I would listen to the brief meditations, and after that I would become calm and ready for the next class."<br>"I loved the breathing space meditation, you can do it anywhere, anytime, it is portable and brings you in the present moment instantly and ."
4.Challenges during practice<br>4.1. Resource related<br>4.2. Ability related	Participants identified certain challenges related to resources available and their own ability that made mindfulness practice a little difficult. Resource related challenges for some included academic work, for a few hectic routine and finding a quiet place for meditation. Ability related challenges included completing worksheets which was seen as an added task, sitting still for meditation and	"Coming back from university in the evening, was tiring and then taking out time for meditations along with university assignments made practice a little difficult."<br>"Listening to audios was difficult due to hectic routine, so I listened to them once and then practiced without audio on the go"<br>"Along with home tasks, filling worksheets felt overwhelming and was time consuming. I wouldn't say it was negative but yes workload increased"<br>"Meditations took time and were required to be done in silence. I live in joint family and finding a quiet place was difficult."

5.Facilitators to practice<br>5.1Participant related<br>5.2 MTC delivery related<br>5.3 Facilitator related<br>5.3.1Facilitator's attitude<br>5.3.2 Facilitator's guidance<br>5.4 MTC elements related	The participants identified certain aspects pertaining to themselves, the delivery of the training, the facilitator and the specific elements of the training that facilitated their mindfulness practice and overcoming the difficulties they experienced during the training. Participant related facilitators included changing their self-critical reaction towards not being able to practice, adjusting the home tasks and readings according to their routine and practicing when they were free or in between other activities, setting reminders of mobile for practices, continued attending session even when they felt tired or had not completed tasks and their motivation towards self-development through this training; reminding themselves that this course is for their personal benefit. Participants found aspects of delivery important in supporting their practice including the multiple groups held every week, groups held on weekends, weekly reminders sent for sessions and tasks and the sharing of experiences and listening to others in group format. Participants considered the facilitator's attitude (non-judgmental, non-critical, accepting, accommodating, gentle and encouraging) and guidance (insights on participants' experiences, guidance during struggles and availability after sessions) to play a major part in facilitating their mindfulness practice. The participants reported different elements of MTC to be facilitative of their mindfulness practice. They found the well-structured nature of the course to be helpful by bringing structure and to their practice. The worksheets helped them reflect and think about their experiences and often led to insights that they were not aware of previously. The home tasks helped practically experience what they had learned in sessions and readings. They found the readings self-explanatory and experiential, adding perspective to their understanding of the content. The participants considered the sessions to be most helpful in supporting their mindfulness practice, based on their queries being addressed, providing a safe space, constructive feedback, meditations practiced and the information provided.	"I managed the difficulties I faced in practicing mindfulness by changing my reaction towards me not practicing, which was self-critical and not helping."<br>"My commute from university is 2 hours so I used that time for weekly readings and audios."<br>I did as much as I could, when ever I got free time, like between classes etc"<br>"I think it was more like I got a chance to work on myself and develop myself."<br>"Usually, word course has a negative connotation that you must study and achieve something. This is the first time I'm hearing of a course which is benefiting only me and that really motivated me to continue and practice."<br>"I happily attended sessions even when I had not done the tasks and it helped me stay connected with the course"<br>"I became very self-critical in the beginning of the course during practices, I shared this in the session and the facilitator guided me that everyone has their own experience, if you are not able to do a practice at a given time, then that is your experience, no need to criticize yourself for it. After that I didn't feel much critical towards myself."<br>"I think the instructor connected me to the course. Sometimes you feel like what you're thinking or feeling is a dumb thing. But she would made you feel that no, this is your experience. Whatever you're feeling and saying is important. Your issues are valid. "

Questionnaire S3: Post intervention Survey-Waitlist group

Mindfulness Training Course-Post-intervention survey(Waitlist group)

1. Have you received any therapy or counseling in the last two months after registering for this research?

Yes No

2- If you answered yes to the first question:

What type of therapy or counseling have you received?

3. 3- If you answered yes to the first question:

How many sessions have you taken in the last two months?

4- Have you read about mindfulness in these last two months?

Yes No

5-In the last two months, have you talked to someone about mindfulness?

Yes No

6- If in the last two months you have received information about mindfulness, what was its nature?

From the students in mindfulness group

From teachers

From internet

From books

From taking any other mindfulness course

7-Would you like to take the mindfulness course?

Yes No

Questionnaire S4: Post-intervention Survey- MTC group

1. How easy was it to use zoom for the online sessions?

   1=not at all 5= very much

2. How much interference of technical problems did you have during sessions?
   1=not at all 5= very much

3. What technical problems did you have?

___

___

4. Overall satisfaction with the sessions being conducted in online mode?
   1= Highly dissatisfied 5=Highly satisfied

5. How effective were the sessions?
   0=Not at all 5= very much
6. Was the information provided in sessions understandable?
   0=Not at all 5= very much
7. Was the information provided in sessions relatable?
   0=Not at all 5= very much
8. Were the meditation practices conducted during sessions helpful?
   0=Not at all 5= very much
9. What was helpful for you during the sessions?
   ___

   ___
10. What was not helpful for you during the sessions?

    ___

    ___

11. Any suggestions to improve the sessions?
    ___

    ___

12. How much time did you spend doing mindfulness meditations on average in a day?
    a) 0 minutes
    b) 5 minutes
    c) 10 minutes
    d) 15 minutes
    e) 20 minutes
    f) 25 minutes
    g) More than 25 minutes
13. Which mindfulness meditations did you find beneficial?
    a) Breath and body meditation (track 1 or 4)
    b) Body scan (track 2)
    c) Mindful movement (track 3)
    d) Sounds and thoughts meditation (track 5)
    e) Breathing space (track 8)
    f) Exploring difficulties meditation (track 6)
    g) Befriending meditation (track 7)

14. How much beneficial were the informal mindfulness activities(other than audio recordings) given to you every week.

    1=Not at all 5= very much
15. Which mindful activity did you find to be the most beneficial?
    a) Doing everyday activities mindfuly
    b) Ten fingers-gratitude list
    c) Keeping the body in mind while talking and listening
    d) Using anchor(feet, breath etc) to ground yourself
    e) Habit releasers
    f) Breathing space
    g) Mindful walking
    h) Practicing kindness towards self
    i) Practicing kindness towards others
    j) Balancing nourishing and depleting activities

16. How many times did you engage in mindfulness activities given as home tasks on average in a day?
    a) 1-2 times
    b) 3-4 times
    c) 5 times
    d) More than 5 times
17. How helpful were the worksheets?

    1=Not at all 5=very much

18. How would you rate the audio recordings?
    a. Not Understandable
       Understandable

0
5

19. How would you rate the weekly chapters?
    a. Not Meaningful
       Meaningful
       0 5
    b. Not Understandable
       Understandable

       0 5

20. How many chapters (from the reading material for every week), did you read? 0-8

---

25. Was the Mindfulness Training Course relevant for you?

0=not at all
5=very much

26. Was the Mindfulness Training Course relevant to stress?

0=not at all
5=very much

27. Was the Mindfulness Training Course relevant to your academic life?

0=not at all
5=very much

Document S5: Semi-structured interview guide to explore the acceptability of students for MTC

The statements in bullet form are to be used as probes.

1.How would you rate your overall satisfaction from the adapted MBI course?(have a number on a scale of 0-10 and a few words to express their level of satisfaction or dissatisfaction)

2.Please share your views about the different mindfulness meditations and home tasks used in the course (could use the probes for each individually; breath and body meditation, body scan, exploring difficulties, sounds and thoughts meditation, 3 minute breathing space,mindful movement meditation,befriending meditation/ habit releasers, 10 finger gratitude list, random acts of kindness, mindful walk, raisin meditation, mindful eating, mindful activities, mindful talking)

What was the experience

How did you feel before doing them and after doing them

What purpose did they serve

3.Please share your views about the group format of the course?

4.Please share your views about the home practice tasks for every week?

What was your experience

How did you manage with your daily routine

5. What during this entire course did you find supporting your engagement and practice?

6. What during this entire course did you find holding you back from engaging and practicing?

How did you overcome

7.What changes did you feel within you? Could you explain please.

Any changes in your behavior, the way you respond, feel, express, think about others and yourself

8. What changes did you feel in your relationships. Could you explain please

Family, friends, work, university, peers, teachers, colleagues, staff, random acquaintances

Any changes in your communication

Any changes in others' responses

9.What did you like most about the Mindfulness Training Course? (Try asking about the content as well as the way of delivery, the process, what made them connect)..If they mention one thing like the readings then you may ask what about the readings did you like, the in text examples, the manner in which it was described (relevance) etc.

Anything in the reading material, audio recordings, online sessions.

10.What did you dislike most about the Mindfulness Training? Same as above

11.What advantages did you experience during and after the course? Further ask if they can identify what in the entire course brought that benefit.

What did you gain from participating in the course

12. What disadvantages did you experience during and after the course? Further ask if they can identify what in the entire course brought that disadvantage.

13. Any adverse effects you experienced from participating in the course?

14. Is there anything else you would like to say about the Mindfulness Training Course?
